# Supplementary material for: Accelerometric Assessment of Postural Balance in Children: A Systematic Review
Source: Diagnostics (Basel). 2020 Dec 22;11(1):8. doi: 10.3390/diagnostics11010008 (PMC7822105; doi:10.3390/diagnostics11010008)
Supplement: Supplementary file 1 [file diagnostics-11-00008-s001.pdf]

**Table S1.** Quality assessment of included articles						
**Quality Index Item**	**Brett et al.[31] (2018)**	**Cheng et al.[38] (2019)**	**Crossley et al.[39] (2018)**	**Fong et al.[45] (2015)**	**García-Liñeira et al.[46] (2020)**	**García-Soidán et al.[47] (2020)**
Were the research objectives or aims clearly stated?	2	2	2	2	2	2
Was the study design clearly described?	0	2	0	2	2	2
Was the study population adequately described?	2	2	2	2	2	2
Were the eligibility criteria specified?	1	2	0	2	2	2
Was the sampling methodology appropriately described?	0	1	0	0	0	0
Was the sample size used justified?	0	2	0	1	0	0
Did the method description enable accurate replication of the measurement procedures?	0	0	2	1	1	2
Was the equipment design and set up clearly described?	0	0	1	2	2	2
Were accelerometers locations accurately and clearly described?	0	2	2	2	2	2
Was accelerometers attachment method clearly described?	0	0	2	1	2	2
Was the signal/data handling described?	1	2	2	2	2	2
Were the main outcomes measured and the related calculations clearly described?	2	2	2	2	2	2
Was the system compared to an acknowledged gold standard?	2	0	0	2	0	0
Were measures of reliability/accuracy of the accelerometers used reported?	0	0	2	0	2	2
Were the main findings of the study stated?	1	0	2	2	2	2
Were the statistical tests appropriate?	2	2	2	2	2	2
Were limitations of the study clearly described?	2	2	2	2	2	2
**Total score** (out of 34)	15	21	25	27	27	28
**Percentage score**	**44.1%**	**61.8%**	**73.5%**	**79.4%**	**79.4%**	**82.4%**
**Quality category**	**Medium**	**Medium**	**High**	**High**	**High**	**High**

Table S1. Quality assessment of included articles (cont.)

Quality Index Item	Iosa, et al.[40] (2018)	Jobbágy et al.[48] (2016)	Kim et al.[43] (2018)	Kim et al.[44] (2018)	Linder et al.[41] (2018)	Mutoh, et al.[32] (2016)	Saether et al.[36] (2015)
Were the research objectives or aims clearly stated?	2	1	2	2	2	2	2
Was the study design clearly described?	1	0	0	0	0	0	1
Was the study population adequately described?	2	1	2	2	2	2	2
Were the eligibility criteria specified?	1	0	2	1	1	2	2
Was the sampling methodology appropriately described?	0	0	2	2	0	0	0
Was the sample size used justified?	0	0	2	2	0	0	0
Did the method description enable accurate replication of the measurement procedures?	0	0	0	1	0	0	0
Was the equipment design and set up clearly described?	2	1	2	2	2	0	1
Were accelerometers locations accurately and clearly described?	2	0	2	2	2	2	2
Was accelerometers attachment method clearly described?	2	1	1	2	2	2	2
Was the signal/data handling described?	2	2	2	2	2	0	2
Were the main outcomes measured and the related calculations clearly described?	2	1	1	1	2	1	1
Was the system compared to an acknowledged gold standard?	0	0	1	1	2	0	1
Were measures of reliability/accuracy of the accelerometers used reported?	0	0	0	0	2	0	1
Were the main findings of the study stated?	2	1	2	2	0	2	2
Were the statistical tests appropriate?	2	0	1	2	2	1	2
Were limitations of the study clearly described?	1	0	2	2	2	2	1
**Total score** (out of 34)	21	8	25	26	23	16	22
**Percentage score**	**61.8%**	**23.5%**	**73.5%**	**76.5%**	**67.6%**	**47.1%**	**64.7%**
**Quality category**	**Medium**	**Low**	**High**	**High**	**High**	**Medium**	**Medium**

**Table S1.** Quality assessment of included articles (cont.).					
**Quality Index Item**	**Shiratori et al.[42] (2016)**	**Speedtsberg et al.[33] (2018)**	**Summa et al.[37] (2015)**	**Tramontano et al.[34] (2017)**	**Wolter et al.[35] (2019)**
Were the research objectives or aims clearly stated?	2	2	2	2	2
Was the study design clearly described?	0	0	0	0	0
Was the study population adequately described?	2	0	1	0	1
Were the eligibility criteria specified?	1	2	0	2	1
Was the sampling methodology appropriately described?	0	0	0	0	0
Was the sample size used justified?	0	0	0	0	1
Did the method description enable accurate replication of the measurement procedures?	1	0	1	0	1
Was the equipment design and set up clearly described?	2	1	2	1	1
Were accelerometers locations accurately and clearly described?	2	2	2	2	2
Was accelerometers attachment method clearly described?	2	0	2	0	2
Was the signal/data handling described?	2	2	2	0	0
Were the main outcomes measured and the related calculations clearly described?	2	2	2	2	2
Was the system compared to an acknowledged gold standard?	2	0	0	2	0
Were measures of reliability/accuracy of the accelerometers used reported?	0	0	0	0	0
Were the main findings of the study stated?	0	2	2	2	1
Were the statistical tests appropriate?	2	2	2	2	2
Were limitations of the study clearly described?	2	2	0	2	0
**Total score** (out of 34)	23	17	18	17	16
**Percentage score**	**67.6%**	**44.1%**	**52.9%**	**50%**	**47%**
**Quality category**	**High**	**Medium**	**Medium**	**Medium**	**Medium**
